# Projecting Thailand physician supplies between 2012 and 2030: application of cohort approaches

**DOI:** 10.1186/1478-4491-11-3

**Published:** 2013-02-01

**Authors:** Rapeepong Suphanchaimat, Thunthita Wisaijohn, Noppakun Thammathacharee, Viroj Tangcharoensathien

**Affiliations:** 1 International Health Policy Program, Ministry of Public Health, Tiwanon Road, Nonthaburi, 11000, Thailand; 2 Health Insurance Systems Research Office, Ministry of Public Health, Tiwanon Road, Nonthaburi, 11000, Thailand

**Keywords:** Cohort analysis, Annual loss rate, Physician projection, Physician to population ratio

## Abstract

**Background:**

This study forecasts physician supply between 2012 and 2030 using cohort analysis, based on future production capacity and losses from the profession, and assesses if, and by when, the projected numbers of physicians would meet the targets of one doctor per 1,500 population, as proposed by the 7^th^ National Conference on Medical Education in 2001, and one per 1,800, proposed by the Ministry of Public Health (MoPH) in 2004.

**Methods:**

We estimated the annual loss rate that best reflected the dynamics of existing practising doctors, then applied this rate to the existing physicians, plus the newly licensed physicians flowing into the pool over the next two decades (from 2012 to 2030). Finally, the remaining practising physicians, after adjustment for losses, were verified against demand projections in order to identify supply gaps.

**Results:**

Thailand has been experiencing an expansion in the total number of physicians, with an annual loss rate of 1%. Considering future plans for admission of medical students, the number of licensed physicians flowing into the pool should reach 2,592 per annum, and 2,661 per annum, by 2019 and 2030 respectively. By applying the 1% loss rate to the existing, and future newly licensed, physicians, there are forecast to be around 40,000 physicians in active clinical service by 2016, and in excess of 60,000 by 2028.

**Conclusion:**

This supply forecast, given various assumptions, would meet the targets outlined above, of one doctor per 1,800 population, and one per 1,500 population, by 2016 and 2020 respectively. However, rapid changes in the contextual environment, e.g. economic demand, physician demographics, and disease burden, may mean that the annual loss rate of 1% used in this projection is not accurate in the future. To ensure population health needs are met, parallel policies on physician production encompassing both qualitative and quantitative aspects should be in place. Improved, up-to-date information and establishment of a physician cohort study are recommended.

## Background

The health workforce is one of six key health system components but is often neglected in developing countries [1] when determining success in achieving national health goals, in particular the Millennium Development Goals (MDGs) [2-4]. A well-functioning health system requires an adequate number of capable, motivated and well-supported health workers; policy makers often ask how many physicians they should produce in order to meet future health needs [5]; accurate predictions of demand for health workers facilitate better production plans.

In the early 1970s, the World Health Organization (WHO) suggested an optimal density of one physician per 5,000 population for developing countries: this ratio was not useful for physician production planning because a country’s level of socio-economic development and the nature of their health-care delivery systems determine the demand for health workers [6]. For instance, in 1992, despite the fact that Thailand had one physician per 4,500 population, well above this benchmark, there were shortages in some areas and maldistribution as a consequence of rapid economic growth that increased demand for doctors in the private health sector in the period from 1992 to 1997 [7-9].

Efforts to forecast demand for, and supply of, health workers, especially physicians, have been ongoing in Thailand since the 1970s [7,10,11]. The summary in Table 1 shows that most studies focused on demand-side projection using service targets or ratios of health workforce per population, with fewer studies applying supply projection.

**Table 1:** Studies of demand for and supply of physicians in Thailand from 1972 to 2004

**Year**	**Organizations**	**Methodology**	**Key results and recommendations**
Before 1972	National Economic and Social Development Board	Physician to population ratio	Need more physicians to meet the target of one doctor to 5,000 population
1979	Coordinating Committee for Medical and Health Affairs	Service targeted approach	Thailand needs 200 more doctors per annum to meet the demand for rural health development project.
1986	The Thai Medical Council	Service targeted approach	Predict a shortage of 4,286 physicians by 2000
1986	National Economic and Social Development Board	Trend projection	Adequate supply by the year 2000
1992	Health Planning Division, the Ministry of Public Health (MoPH)	Physician to population ratio	Increase production in response to increased demand due to economic growth, need 340 more doctors per annum
1994	Bureau of Health Policy and Planning, the MoPH	Combination of physician to population ratio, service targeted and health demand approach	Increase production to fill in the expanded rural health services. A Collaborative Project to Increase Production of Rural Doctors was decided by the Cabinet to produce 300 more doctors per year
1995-1996	Bureau of Health Policy and Planning, Praboromrachanok Institute for Human Resource Development, the MoPH and Health Systems Research Institute	Combination of physician to population ratio and service targeted and health demand approach	Adequate supply by 2015
1996	Thailand Development Research Institute	Health demand approach	Adequate supply by 2020
1996	Bureau of Policy and Strategy, the MoPH	Cohort analysis, annual loss rate method and modified physician to population ratio	The supply of physicians by the year 2020 will be 44,028 (using high-loss scenario) to 47,519 (using low-loss scenario)
2001	The 7th National Conference on Medical Education	Health demand approach and physician to population ratio	Increase production to reach an optimal ratio of one physician to 1,500 population by 2021
2004	Bureau of Policy and Strategy, the MoPH	Physician to population ratio	Increase production to reach an optimal ratio of one physician to 1,800 population; 6,000 additional more physicians should be produced in 2006

Sources: Tida Suwannakij (1996); Thaksaphol Thammarangsri (2005).

Among the few studies that did examine supply projection, Suwannakij et al., in 1996 [7], applied cohort analysis and the annual loss rate method. A number of assumptions of physician annual loss rate were applied: <2.5% in an expanding state, >2.5% in a shrinking state and 2.5% for equilibrium state [12]. The 2.5% figure was estimated from the assumption that a physician’s loss from clinical practice would be 100% after 40 years of working, thus the annual loss rate would be 100/40 = 2.5%. The projection appears to have been fairly accurate in projecting the actual number of physicians in the first few years following the study, however, no subsequent studies applied this method and verified whether the forecast of supply matched the demand for physicians as proposed by numerous organizations, as is presented in Table 1 above [13,14].

To fill the gap, this study forecasts physician supply between 2012 and 2030 based on future production capacity and losses from the profession, and assesses whether, and if so when, the projected supplies will meet the respective goals of one doctor per 1,500 population as proposed by the 7^th^ National Conference on Medical Education in 2001, and one per 1,800 population as proposed by the Ministry of Public Health (MoPH) in 2004.

## Material and method

Among the three types of projection tools available (cohort, observed change and two life-table methods) [15], this study applies the cohort method. Health workforce loss rate is commonly applied in supply projection, especially in cohort and life-table approaches.

**Figure 1:**
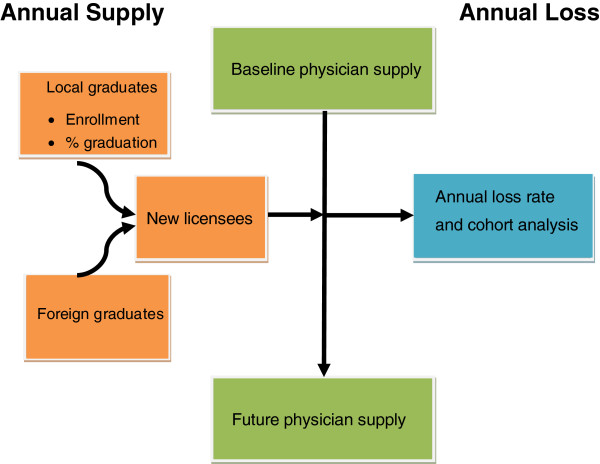
Framework of the dynamics of physician supply.

The following approaches were utilised: we estimated the value of an annual loss rate that best reflected the dynamics of existing practising doctors and this loss rate was applied to all existing physicians in the pool, as well as newly licensed physicians flowing into the pool over the next two decades (from 2012 to 2030). Finally, the supply projection of practising physicians adjusted for losses was verified against the demand projection using one per 1,500, and 1,800 population, in order to identify supply gaps See Figure 1.

In making these projections, we required (a) accurate numbers of the existing pool of practising doctors (stock), (b) the annual number of licensed physicians entering into the pool, both domestic and foreign graduates (flow), minus (c) losses from the profession through death, retirement and leaving clinical practice to take up alternative roles e.g. administration, or research, or leaving the profession entirely. Projections of the supply of practising physicians are reliable when these data are available and accurate [15].

This study applied the assumptions presented in Suwannakij [7] but with new data. Age-specific loss rates was solicited from the opinions of three experts, these were two senior officers within the MoPH and the director of the Health Systems Research Institute [7]. At the time of writing they posited that, in an expanding state, the annual loss rate among 25- to 59-year-olds would be 0.15% to 0.6% (averaging 0.45%) with this annual loss rate, at the end of 35 working years (assuming entry to the workforce at 24 years old), one year before the mandatory retirement age of 60 years old, there would be 81–95% of physicians still pursuing a medical career. At the retirement age of 60 years old, the experts forecasted an annual loss rate of 40%, while during the first 4 years after reaching 60 years old the annual loss rate would decrease to 10%. This would then rebound to 30% annual loss for the remainder of the working period after 64 years old. This meant that only 44% of physicians between 61 and 70 years old would still be engaged in clinical practice, and very few would remain in clinical services after 70 years old.

### Flow of future licensed physicians

The number of domestic and foreign graduates gaining a licence to practise was retrieved from the Thai Medical Council. There are 18 public medical schools, and one private medical school, in Thailand. All domestic medical graduates have to pass the comprehensive examination, held by their respective schools, and must also pass the National Licensing Examination, held by the Thai Medical Council, in order to be licensed to practise in Thailand.

Likewise, all foreign medical graduates are required to hold a diploma from a medical school recognized by the Thai Medical Council and must hold a licence to practise in that country [16] prior to applying to sit the National Licensing Examination held by the Council. On passing that exam they can then gain a licence to practice in Thailand.

### Graduates who pass the licensing examination

The numbers of local graduates gaining licences was estimated from the rate of graduates who passed the National Licensing Examination multiplied with the number of planned future admissions at all 19 medical schools. Currently, there are no explicit plans from the MoPH or, known private institutes, to open new medical schools in the near future. The number of medical schools used in this study is thus 19.

The historical numbers of medical student admissions were retrieved from the Human Resources for Health Research and Development Office (HRDO), at the MoPH, while the numbers of newly licensed doctors were retrieved from the Thai Medical Council. For newly established medical schools where students have not yet graduated, the average graduation and licensing rate of all other medical schools were applied. The data on the future admission plans of each medical school were retrieved from the HRDO.

This study assumes no significant changes in the number of licensed foreign graduates in the next two decades. The average number of licensed doctors, graduating from overseas, is applied at the same rate for the future as was seen in the past two decades.

### Stock of existing physicians and annual loss rate

Stock data on existing licensed doctors between 1937 and 2010 was retrieved from the Thai Medical Council. This dataset counts the registration number, which is unique to each physician, ensuring no double counting. However, this dataset counts all registered physicians regardless of whether they are in active professional service or not, and physicians who have died are not de-registered. We devised methods to clean this dataset with the aim of estimating the number of physicians in active clinical practice.

The Medical Council conducted two surveys of physicians in 2010 and 2011. The surveys reported 37,396 and 39,269 physicians, subdivided by age profile, who were able to be contacted by mail, but unfortunately no information on their current professional practice: see Table 2. We modified this information, the only source that is available, by assuming that all contactable physicians aged 20 to 60 years old, and 44% of physicians 60 to 70 years old, were active in professional life either in clinical service, research, teaching or administration. However, all physicians more than 70 years old were considered inactive. We then calculated the proportion of active physicians to total registered numbers as 83% in 2010 and 2011.

**Table 2:** The number of physicians categorized by age group and proportion of active physicians to total registered physicians between 2010 and 2011

**Number of physicians**	**2010**	**2011**
a) Age 20-30	9,865	10,234
b) Age 31-40	10,553	11,292
c) Age 41-50	7,386	7,620
d) Age 51-60	4,959	5,202
e) Age 61-70	2,347	2,543
f) Age more than 70	2,178	2,280
g) Unknown age	108	98
Total contactable physicians	37,396	39,269
Estimated total active physicians^a^	33,937	35,620
Total registered physicians at the Council	41,015	42,890
Proportion of active physicians to total registered physicians	83%	83%

^a^Total active physicians = a + b + c + d + (e x 44/100).

Source: The Thai Medical Council (2011).

The calculated figure of 83% was used to reduce the total number of registered physicians, in the years 2000 to 2011, to leave the number of active professionals. Further, a study by Thammarangsri in 2005 showed around 8% of total active physicians did not engage in clinical practice [10]; this figure was applied to scale down the number of active physicians to only those who were actively engaging in clinical services, see the final column of Table 3.

**Table 3:** Total registered physicians, estimated active and clinical practising physicians between 2000 and 2011

**Year**	**Total registered numbers**	**Total active physicians (83% of registered number)**	**Total physicians in clinical practice (92% total active physicians^a^)**
2000	26,226	21,768	19,972
2001	27,498	22,823	20,940
2002	28,824	23,924	21,950
2003	30,300	25,149	23,074
2004	31,730	26,336	24,163
2005	33,280	27,622	25,344
2006	34,820	28,901	26,516
2007	36,392	30,205	27,713
2008	37,841	31,408	28,817
2009	39,204	32,539	29,855
2010	41,015	34,042	31,234
2011	42,890	35,599	32,662

**^a^**Adjusted from Thaksaphol Thammarangsri (2005).

### Fine-tuning the annual loss rate

In the last decade, an increasing loss of physicians from the profession has been observed [10,17,18]; the assumed annual loss rate of 0.45% appears to be too low and should, therefore, be re-estimated. The annual loss rate required adjustment to achieve the best fit with the number of active physicians as produced in the final column of Table 3. By gradually increasing the annual loss rate, starting from 0.45%, we found that a 1% annual loss rate best fitted the number of active physicians in the final column of Table 3.

**Figure 2:**
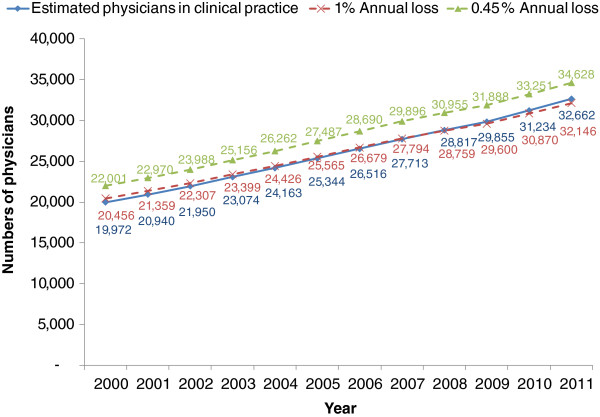
Comparing estimated active physicians with the results from a 0.45% and a 1% annual loss rate.

In Figure 2 when a 0.45% annual loss rate was applied, there was a 7% overestimate of the total number of active physicians. However, when a 1% annual loss rate was applied, it closely matched the estimated number of active physicians, see Figure 2. Therefore, we decided to apply a 1% annual loss rate in the projection between 2012 and 2030.

## Results

### Flow of future licensed physicians

**Figure 3:**
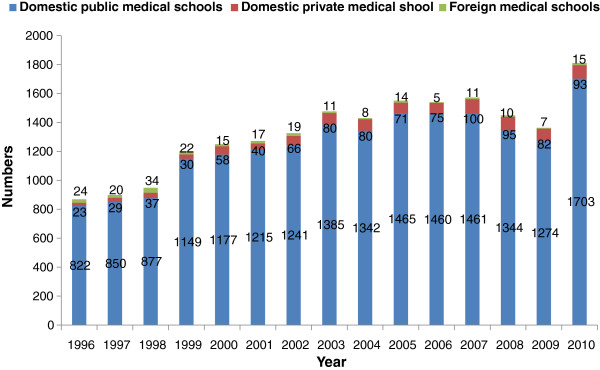
Medical graduates gaining licences from public and private domestic and foreign medical schools between 1996 and 2010.

Figure 3 clearly demonstrates the dominant contribution of domestic public medical schools. In 2010, there were approximately 1,800 graduates gaining licences in public schools: double the number in 1996. This was a result of a rapid expansion in the number of public medical schools, from 13 to 18 over the past 15 years. Also, the one existing private medical school tripled their production capacity, from 30 graduates per year in 1999, to 93 graduates in 2010. Licensed physicians from foreign institutions played a negligible role, averaging 15 per annum. There was a dip in the number of licensed doctors from 2007 to 2009 as a result of a reduction in admissions in the 2002 and 2003 student cohorts in response to budget reductions; this rebounded from 2004 onwards as a Cabinet Resolution endorsed the increased production of doctors [19], see Figure 3.

Table 4 provides the pass rates of medical graduates taking the national licensing examination in 19 schools: ranging from 90.6% in PMK to 99.4% in VJ. The rate from individual schools was applied to Table 5 to estimate the total number of medical graduates gaining licences between 2012 and 2030. With this future admission plan, the flow of future licensed physicians into the pool would exceed 2,500 per year from 2017 onwards See Table 5.

**Table 4:** Rates of graduates who passed the National Licensing Examination for each medical school in Thailand

**Medical school**	**Years of admission**	**Total admissions**	**Total graduates who passed the National Licensing Exam**	**Years of graduation**	**Rates of graduates who passed the National Licensing Exam**
Vajira (VJ)	2001-2004	161	160	2007-2010	99.4
Khon Kaen (KK)	2001-2004	599	589	2007-2010	98.3
Chiang Mai (CMU)	2001-2004	664	655	2007-2010	98.6
Thammasat (TU)	2001-2004	348	330	2007-2010	94.8
Princess of Naradhiwas (PNU)	2007-2011	88	-	2013-2017	-
Naresuan (NSU)	2003-2004	292	279	2009-2010	95.5
Burapha (BU)	2007-2010	144	-	2013-2016	-
Chulalongkorn (CU)	2001-2004	869	846	2007-2010	97.4
Phayao (PU)	2011	15	-	2017	-
Phramongkutklao (PMK)	2001-2004	192	174	2007-2010	90.6
Mahasarakham (MSU)	2007-2010	249	-	2013-2016	-
Ramathibodi (RA)	2000-2004	505	494	2006-2010	97.8
Walailak (WA)	2008-2010	143	-	2014-2016	-
Srinakharinwirot (SWU)	2000-2003	390	376	2006-2009	96.4
Siriraj (SI)	2000-2003	798	791	2006-2009	99.1
Prince of Songkla (PSU)	2001-2003	583	553	2007-2009	94.9
Suranaree University of Technology (SUT)	2006-2010	240	-	2012-2016	-
Ubonratchathani (UBU)	2006-2010	134	-	2012-2016	-
Rangsit (RSU)^a^	2000-2004	456	445	2006-2010	97.6
**Total**		5,857	5,692		97.2

**^a^**Faculty of Medicine, Rangsit University, is the only private medical school in Thailand.

Source: Human Resources for Health Research and Development Office (HRDO), MoPH (2011).

**Table 5:** Future admission plans and projected annual graduates by 19 domestic medical schools and annual foreign graduates

**Medical school**	**Graduation percentage**	**Graduates per year**	**2012**	**2013**	**2014**	**2015**	**2016**	**2017**	**2018**	**2019**	**2020**	**2021**	**2022**	**2023**	**2024**	**2025**	**2026**	**2027**	**2028**	**2029**	**2030**
VJ	99.4%	Admissions	80	80	80	80	80	80	80	80	80	80	80	80	80	80	80	80	80	80	80
		Graduates gaining licence	80	80	80	80	80	80	80	80	80	80	80	80	80	80	80	80	80	80	80
KK	98.3%	Admissions	247	285	281	275	283	288	288	288	288	288	288	288	288	288	288	288	288	288	288
		Graduates gaining licence	243	280	276	270	278	283	283	283	283	283	283	283	283	283	283	283	283	283	283
CMU	98.6%	Admissions	188	188	232	251	249	250	250	250	250	250	250	250	250	250	250	250	250	250	250
		Graduates gaining licence	185	185	229	248	246	247	247	247	247	247	247	247	247	247	247	247	247	247	247
TU	94.8%	Admissions	134	131	156	161	187	177	177	177	177	177	177	177	177	177	177	177	177	177	177
		Graduates gaining licence	127	124	148	153	177	168	168	168	168	168	168	168	168	168	168	168	168	168	168
PNU	97.2%	Admissions	0	0	16	24	24	24	24	24	24	36	36	36	36	48	48	48	48	48	48
		Graduates gaining licence	0	0	16	23	23	23	23	23	23	35	35	35	35	47	47	47	47	47	47
NSU	95.5%	Admissions	168	128	151	136	172	169	165	165	165	180	180	180	180	180	180	180	180	180	180
		Graduates gaining licence	161	122	144	130	164	161	158	158	158	172	172	172	172	172	172	172	172	172	172
BU	97.2%	Admissions	0	0	32	32	32	48	48	48	48	48	48	48	48	48	48	48	48	48	48
		Graduates gaining licence	0	0	31	31	31	47	47	47	47	47	47	47	47	47	47	47	47	47	47
CU	97.4%	Admissions	248	278	273	291	302	313	313	313	313	313	313	313	313	313	313	313	313	313	313
		Graduates gaining licence	241	271	266	283	294	305	305	305	305	305	305	305	305	305	305	305	305	305	305
PU	97.2%	Admissions	0	0	0	0	0	0	15	30	30	30	30	30	30	30	30	30	30	30	30
		Graduates gaining licence	0	0	0	0	0	0	15	29	29	29	29	29	29	29	29	29	29	29	29
PMK	90.6%	Admissions	100	100	100	100	100	100	100	100	100	100	100	100	100	100	100	100	100	100	100
		Graduates gaining licence	91	91	91	91	91	91	91	91	91	91	91	91	91	91	91	91	91	91	91
MSU	97.2%	Admissions	0	48	48	48	48	50	50	50	50	50	50	50	50	50	50	50	50	50	50
		Graduates gaining licence	0	47	47	47	47	49	49	49	49	49	49	49	49	49	49	49	49	49	49
RA	97.8%	Admissions	128	131	158	160	161	180	180	180	180	180	180	180	180	180	180	180	180	180	180
		Graduates gaining licence	125	128	155	157	157	176	176	176	176	176	176	176	176	176	176	176	176	176	176
WU	97.2%	Admissions	0	0	0	48	47	48	48	48	48	48	48	48	48	60	60	60	60	60	60
		Graduates gaining licence	0	0	0	47	46	47	47	47	47	47	47	47	47	58	58	58	58	58	58
SWU	96.4%	Admissions	120	120	120	120	120	150	165	180	180	180	180	180	180	180	180	180	180	180	180
		Graduates gaining licence	116	116	116	116	116	145	159	174	174	174	174	174	174	174	174	174	174	174	174
SI	99.1%	Admissions	242	232	233	247	291	292	292	292	292	292	292	292	292	292	292	292	292	292	292
		Graduates gaining licence	240	230	231	245	288	289	289	289	289	289	289	289	289	289	289	289	289	289	289
PSU	94.9%	Admissions	175	185	188	183	195	200	200	200	200	200	200	200	200	200	200	200	200	200	200
		Graduates gaining licence	166	175	178	174	185	190	190	190	190	190	190	190	190	190	190	190	190	190	190
SUT	97.2%	Admissions	0	48	48	48	49	48	60	60	60	80	80	80	80	80	80	80	80	80	80
		Graduates gaining licence	0	47	47	47	48	47	58	58	58	78	78	78	78	78	78	78	78	78	78
UBU	97.2%	Admissions	0	50	16	16	16	50	50	50	50	50	50	50	50	50	50	50	50	50	50
		Graduates gaining licence	0	49	16	16	16	49	49	49	49	49	49	49	49	49	49	49	49	49	49
RSU	97.6%	Admissions	100	108	113	137	129	120	120	120	120	120	120	120	120	120	120	120	120	120	120
		Graduates gaining licence	98	105	110	134	126	117	117	117	117	117	117	117	117	117	117	117	117	117	117
Total number of graduates gaining licences			1872	2050	2179	2288	2412	2511	2548	2577	2577	2623	2623	2623	2623	2646	2646	2646	2646	2646	2646
Average number of foreign-trained graduates gaining licences			15	15	15	15	15	15	15	15	15	15	15	15	15	15	15	15	15	15	15
**Total number of graduates gaining licences**			**1887**	**2065**	**2194**	**2303**	**2427**	**2526**	**2563**	**2592**	**2592**	**2638**	**2638**	**2638**	**2638**	**2661**	**2661**	**2661**	**2661**	**2661**	**2661**

Source: The Medical Council of Thailand (2011); Human Resources for Health Research and Development Office (HRDO), MoPH (2011).

### Cohort analysis applying the 1% annual loss rate

Additional file 1: Table S1 provides the full cohort analysis table with the application of the 1% annual loss rate for the existing pool and the new licensed doctors flowing into the pool; See Additional file 1: Table S1. The future supply of physicians, active in clinical practice, would be around 40,000 by 2016 and would exceed 50,000 and 60,000 by 2022 and 2028 respectively.

Table 6 compares the future supply of physicians from the cohort analysis with the estimated population in the corresponding period [20]. With these figures, the doctor density would reach the goals of one doctor per 1,800 population and one per 1,500 population by 2016 and 2020 respectively See Table 6.

**Table 6:** Projecting physician supply and future population between 2012 and 2030

**Year**	**Estimated population (thousands)**^**^a^**^	**Estimated physician supply**	**Physician to population ratio**
2012	68,251	33,471	1: 2,039
2013	68,610	34,944	1: 1,963
2014	68,980	36,515	1: 1,889
2015	69,222	38,168	1: 1,814
**2016**	**69,455**	**39,909**	**1: 1,740**
2017	69,679	41,718	1: 1,670
2018	69,893	43,540	1: 1,605
2019	70,100	45,349	1: 1,546
**2020**	**70,217**	**47,081**	**1: 1,491**
2021	70,330	48,762	1: 1,442
2022	70,440	50,505	1: 1,395
2023	70,547	52,197	1: 1,352
2024	70,651	53,864	1: 1,312
2025	72,065	55,505	1: 1,298
2026	72,400	57,101	1: 1,268
2027	72,734	58,666	1: 1,240
2028	73,069	60,204	1: 1,214
2029	73,403	61,717	1: 1,189
2030	73,738	63,212	1: 1,167

**^a^**The rows highlighted indicate years when the national goals of physician to population ratio of 1:1,800 and 1:1,500 will be achieved.

Source: National Economic and Social Development Board (2003).

## Discussion

This study is an extensive attempt at applying the cohort approach in projecting future supply of physicians in Thailand with a 1% annual loss rate. This assumes that the physician population is in an expanding state despite the increase in the annual loss rate in comparison to that found in the previous study by Suwannakij et al. in 1996. Given that the assumptions are valid, especially the 1% annual loss rate, the fine-tuning of the Medical Council dataset on the stock of physicians, the future production capacities of medical schools, and the rate of graduates gaining licences, the projected supplies will be able to match the national goals of one doctor per 1,500 population as proposed by the 7^th^ National Conference on Medical Education by 2020, and one per 1,800 proposed by the MoPH by 2016.

A key factor that has kept the supply of physicians in an expanding state has been the rapid increase in physician production over the past two decades. This has been the result of a set of policies aimed at expanding physician supply, e.g. establishing a number of new public medical schools, recruiting a greater number of students into medical schools, in particular those in remote areas, through various modes of admission. The Collaborative Project to Increase Rural Doctors (CPIRD), launched by the MoPH in 1994, is a good example of these policies. It has enrolled around 300 students per annum from rural backgrounds, with a mandate that these students need to serve their hometown upon graduation [21]. The project also plans to increase its production levels to produce 3,807 graduates between 2009 and 2019.

In carrying out this study, several limitations were addressed with a rigorous evidence-based methodology. However, several remaining limitations were identified as follows:

The assumption that the cohort age-specific loss rate will remain unchanged in the future may not hold true [15], because other elements of the contextual environment may change rapidly, such as, labour market dynamics. Since the recovery from the economic crisis in early 2000, the private sector’s demand for physicians has grown significantly and may cause a brain drain of physicians from the public sector [21]. In the context of the upcoming 2015 inauguration of the ASEAN Economic Community [22], the relaxation of medical council requirements on licensing may increase the number of foreign graduates practising in Thailand; meanwhile, licensing relaxation in recipient countries may stimulate outward migration of Thai physicians to practise abroad. Increased job opportunities in other economic sectors may also stimulate losses from professional practice.

A significant change in the demographics of physicians, specifically the great increase in female students enrolling in medical schools, may affect physicians’ career patterns and as such the loss rate will need further examination. The evidence from a survey of new medical graduates in 2011, by the International Health Policy Program, revealed that only 40% of new graduates were male [23]. This is in contrast to the current gender ratio of overall physicians in Thailand in which around two-thirds of physicians are male [24].

The cleaning of the Thai Medical Council’s dataset on the stock of physicians relied on the 2010 and 2011 surveys, which concluded that 83% of total registered physicians were active in clinical practice; the surveys may have had numerous limitations, such as the representativeness of respondents to the overall physician population, and neither took into account the potential for a return to practice after a career break. This inevitably has an effect on the accuracy of this data.

Focusing on the national average obscures the issue of the sub-national distribution of physicians, for which parallel policies should be in place to address imbalances. This study quantifies the future supply of physicians; however, quality, in terms of clinical competency, communication skills, human interaction, social skills and attitudes and capacity to conduct ‘interprofessional teamwork’, is an equally important factor determining productivity and outcomes. This study indicates that, if the given assumptions hold, current production capacity is adequate to meet the national goals of physician density of 1:1,800 by 2016 and 1:1,500 by 2020, but policy makers should not be complacent as a result of these findings. This supply of physicians, if achieved, does not fully ensure that there will be adequate physicians to respond to the health needs of the population. Maldistribution is still a major policy concern; in 2005, physician density was around 1:800 in Bangkok, almost nine times as high as the density in the Northeast where it was 1:7,000. Almost one quarter of the total number of physicians was employed in the private sector [25] and this trend is increasing [17].

It should be noted that the results of this study will yield greater benefits if integrated into the production and employment planning of other health professions e.g. nurses, dentists, pharmacists, and also medical specialities. Elaborating on the findings of the study, by taking into account the production planning of other professions in concordance with the population’s health needs, may be useful for policy implementation in an effective manner.

Evidence-based health workforce policy and planning requires more accurate data on both stock and flow of physicians. While there has previously been ad hoc physician cohort data collection undertaken, there is a need for regular, routine and integrated collection of physician data by cohort, as part of a strengthening of the whole human resources for health information system. This would improve on the routine registration dataset collected by the Medical Council, ensure greater provision of information to the public, and reflect professional employment dynamics, in particular, losses from the profession and from clinical practice. Ad hoc surveys usually suffer from poor response rates and the non-representativeness of respondents. A 20-year Thai nurse cohort study [26], launched in 2010, provides useful information on the life dynamics of Thai nurses, it monitors the loss rate and assesses the duration of the nursing career – one of the vital parameters in health workforce planning. Lessons from the nurse cohort study would be useful for establishing a physician cohort study in Thailand.

## Conclusion

Despite data limitations, given various assumptions and data cleaning of stock, with the application of the cohort approach and a 1% loss rate for both stock and flow, while assuming future production by all medical schools adheres to the plan, this paper thoroughly estimates the future supply of physicians in Thailand. It clearly shows that the desired physician density of 1:1,800 and 1:1,500 can be achieved by 2016 and 2020 respectively.

Given these assumptions, the current production capacity in Thailand can meet the goals of physician density by 2020. However, this may not fully ensure an adequate supply of physicians to respond to the health needs of the population, nor equitable distribution across provinces, unless other parallel policies are in place. Further studies on physician dynamics based on improved, up-to-date and accurate health workforce information, and the establishment of a doctor cohort study are recommended.

## Competing interests

The authors declare that they have no competing interests.

## Authors’ contributions

Design of the study by RS, TW and NT. Data collection and analyses by RS and VT. Manuscript writing by RS, TW, NT and VT. All authors contributed to the revision of the draft and agreed upon the final manuscript. All authors read and approved the final manuscript.

## Supplementary Material

Additional file 1: Table S1. Full cohort analysis table with 1% annual loss rate. Click here for file
